# Supplementary material for: The RNA polymerase clamp interconverts dynamically among three states and is stabilized in a partly closed state by ppGpp
Source: Nucleic Acids Res. 2018 Jun 6;46(14):7284–95. doi: 10.1093/nar/gky482 (PMC6101503; doi:10.1093/nar/gky482)
Supplement: Supplementary Data [file gky482_supplemental_figures.doc]

**Supplementary Information for the manuscript:**

**The RNA polymerase clamp interconverts dynamically among three states and is stabilized in a partly closed state by ppGpp**

Diego Duchi, Abhishek Mazumder, Anssi M. Malinen, Richard H. Ebright and Achillefs N. Kapanidis

**SUPPLEMENTARY FIGURE LEGENDS**

**Fig. S1** **A** An example of the field of view for typical coverage seen for RNAP non-specifically adsorbed to a PEG functionalised glass surface (left), an example view of the typical coverage seen for RNAP specifically immobilized on a similar surface via penta-His antibody/his-tag interactions (right). The left panel shows the donor channel, the right panel shows the acceptor channel. Scale bar, 5 μm**. B** Bar graph with average number of fluorescent molecules per imaging due to specific immobilisation and non-specific adsorption. Error bars represent the standard deviation across 5 images. **C** Accurate FRET (E_a_) histogram for all molecules before filtering of data according to procedures described in Materials and Methods. **D** Representative examples of traces which exhibit unusually high fluorescence (possibly aggregates of RNAP; top) or multiple photo-bleaching steps (bottom).

**Fig. S2 A** Uncorrected FRET efficiency (E*) histograms generated from HMM fits of the data to two- to six- state analysis in ebFRET for an in-vitro reconstituted doubly fluorescently labeled His-tagged RNAP. **B** Plot of the mean values for lower bound per series for the models with different number of states as extracted from the analysis. (top) and plot of the values of AIC as calculated for the models with different number of states (bottom). **C** Dwell time distributions for open, partly closed and closed clamp conformations. D Table showing transition rates for all possible transitions as estimated from the HMM fit to the data.

**Fig. S3** **A** Uncorrected FRET efficiency (E*) histogram for an in-vitro reconstituted (top) or an in-vivo reconstituted (bottom) fluorescently labeled RNAP holoenzyme. Grey (all frames), blue (open clamp), green (partially closed clamp) and red (closed clamp) bars are as obtained from a three state HMM fit to the data. **B** Rates of inter-conversion between the three states for an in-vitro reconstituted (top) or an in-vivo reconstituted (bottom) fluorescently labeled RNAP holoenzyme. Data acquisition for the in-vitro reconstituted RNAP was done in T8 buffer at 22^ο^C and for the in-vivo reconstituted RNAP data acquisition was done in KG7 buffer at 22^ο^C. Frame exposure time: 20 ms. **C** Structures of RNAP with accessible volume clouds of FRET probes and predicted mean dye distances between the FRET probes generated from accessible volume calculations. Accesible volume clouds and mean dye positions for Cy3B and Alexa647 are depicted in green and red respectively.

**Fig. S4** **A** Uncorrected FRET efficiency (E*) histograms of an in-vivo reconstituted fluorescently labeled RNAP holoenzyme in presence of increasing concentrations of ppGpp (0 to 1 mM). **B** Example traces showing transition between clamp conformational states in presence of 1mM ppGpp. Data acquisition was done in KG7 buffer at 22^ο^C. Frame exposure time: 20 ms.

**Fig. S5 A** Example time-traces of uncorrected FRET efficiency (E*) for fluorescently labeled RNAP holoenzyme in presence of myxopyronin (20 μM); frame exposure time: 20 ms. **B** Example time traces of fluorescence intensity and E* showing clamp closing upon myxopyronin addition (final concentration of 20 μM) in real time. Frame exposure time: 200 ms. Data acquisition was done in T8 buffer at 22^ο^C.

**Fig. S6** Example time-traces of uncorrected FRET efficiency (E*) of **A** open complex (RP_O_) formed on lacCONS+2 (-39/+25) ds DNA fragment; **B** open complex (RP_O_) formed on a pre-melted lacCONS+2 (-39/+25) ds DNA fragment; **C** elongation complex (RDe) formed on lacCONS-14 (-107/+56) ds DNA fragment. Frame exposure time: 20 ms. Data acquisition was done in T8 buffer at 22^ο^C.

**Fig. S1**

**A**

**
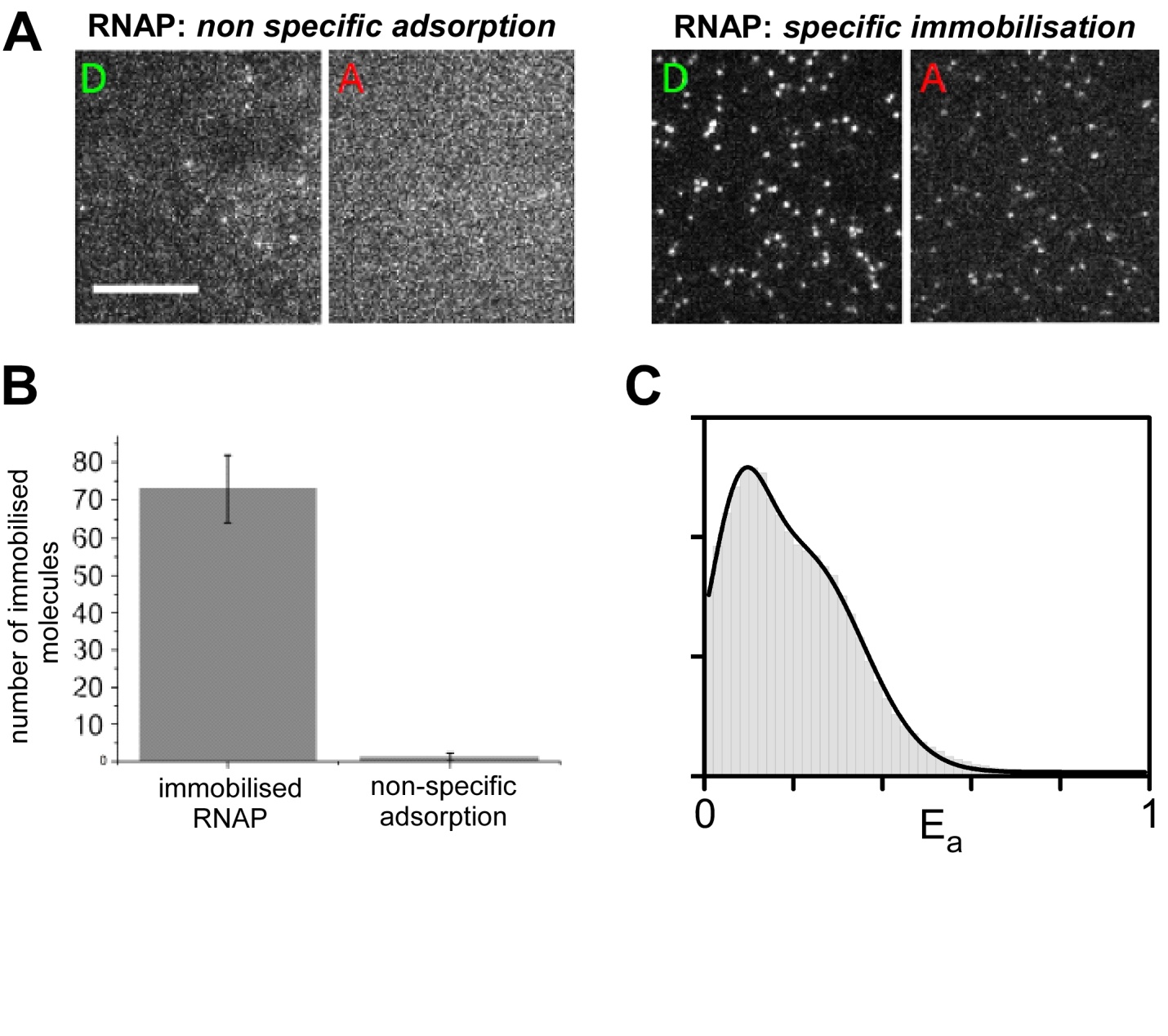
**

**12000**

**B**

**C**


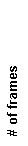


**D**


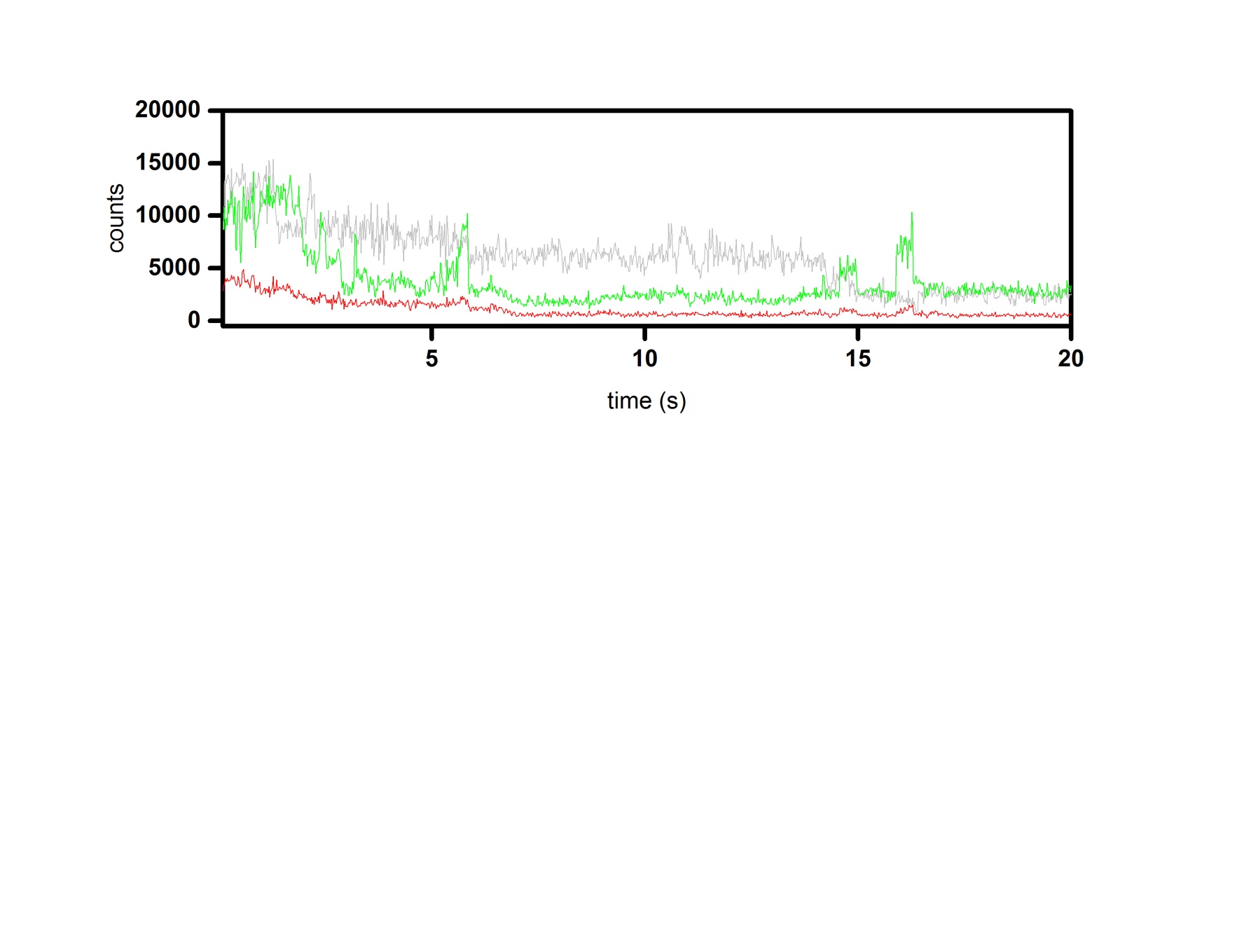
**D**

**very high counts: possible aggregates DDDAAA**

**DDDAAA**

**multiple photobleaching steps**

**multiple photobleaching steps**

**
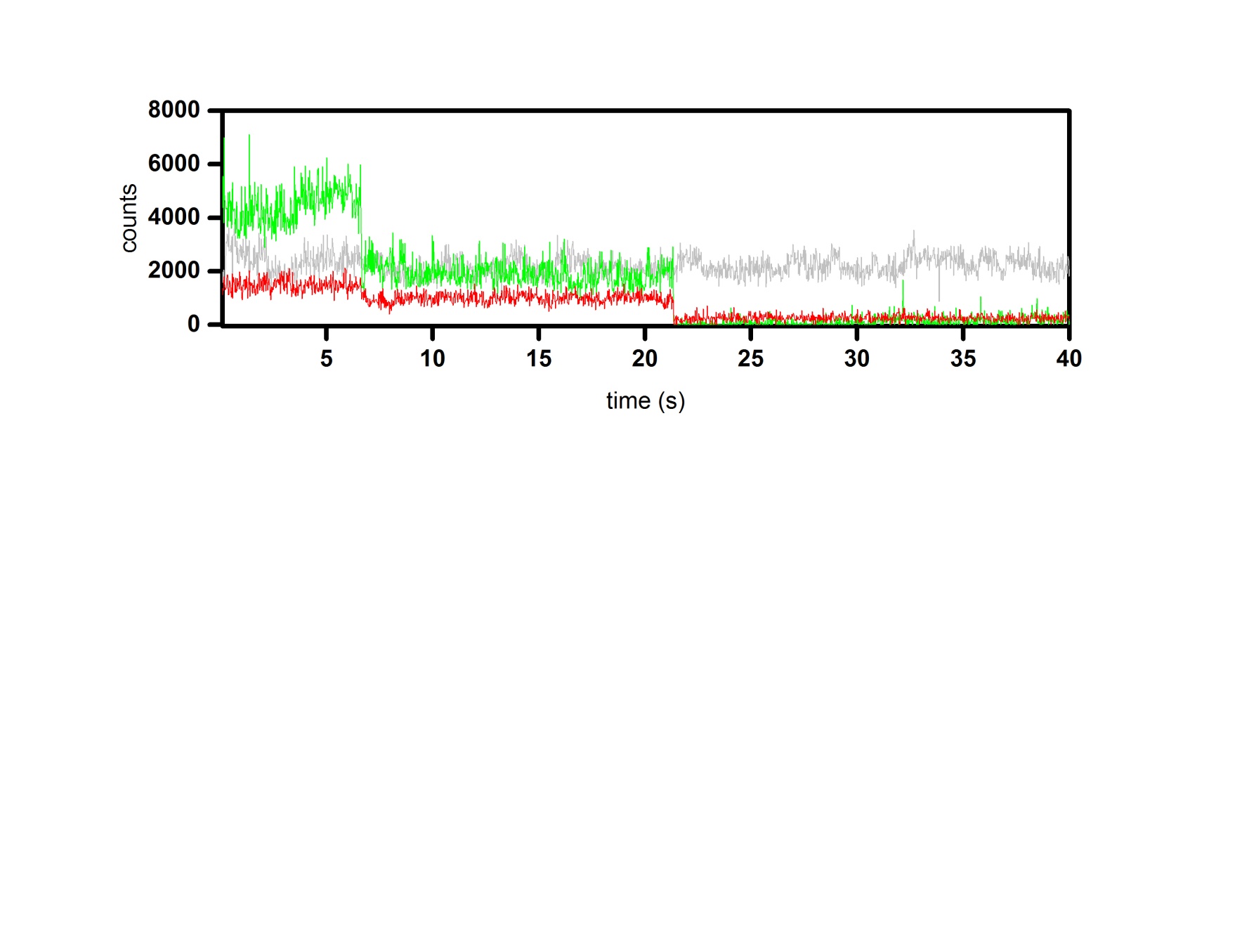
**

**Fig. S2**

**
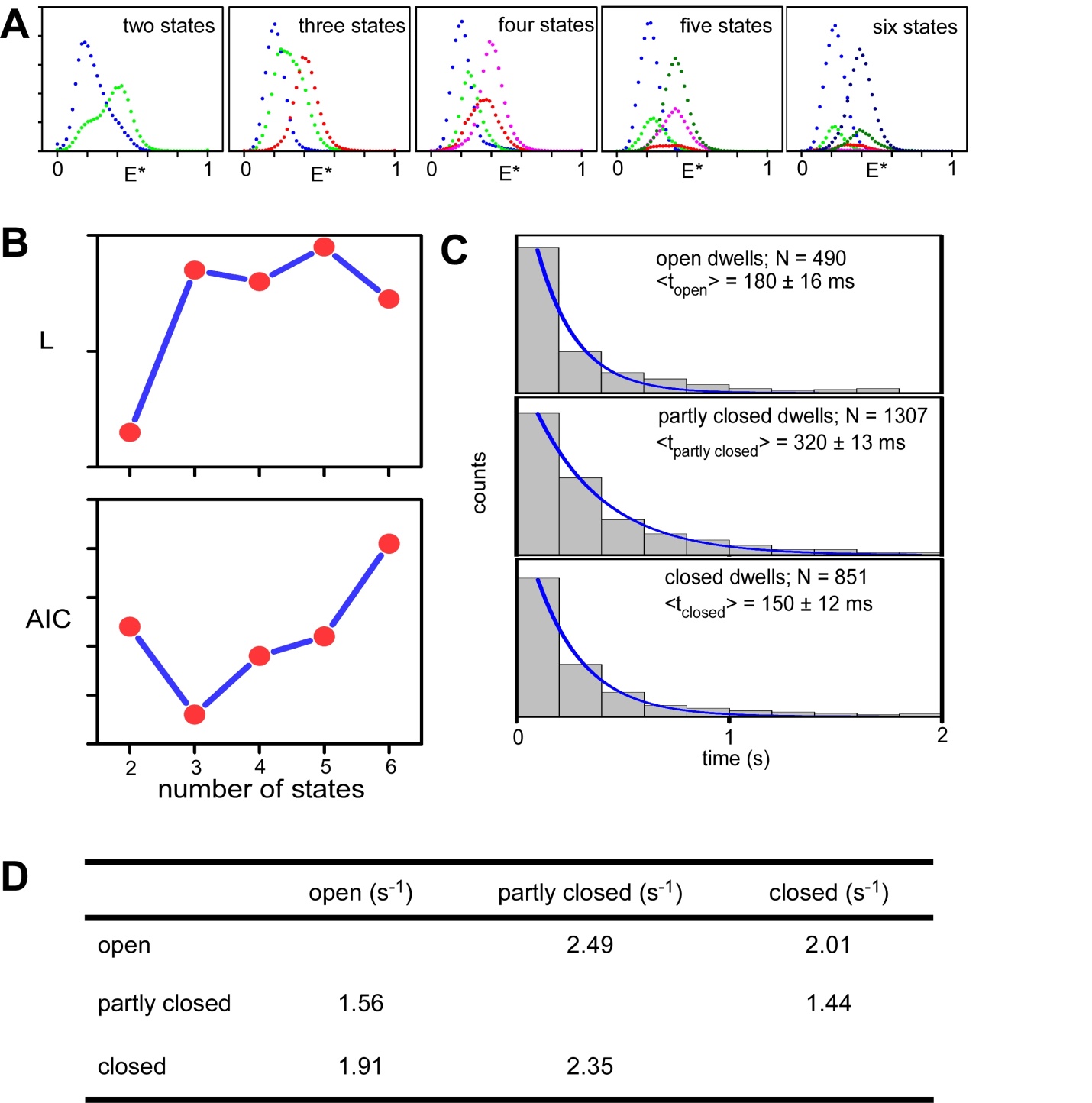
**

**Fig. S3**

**
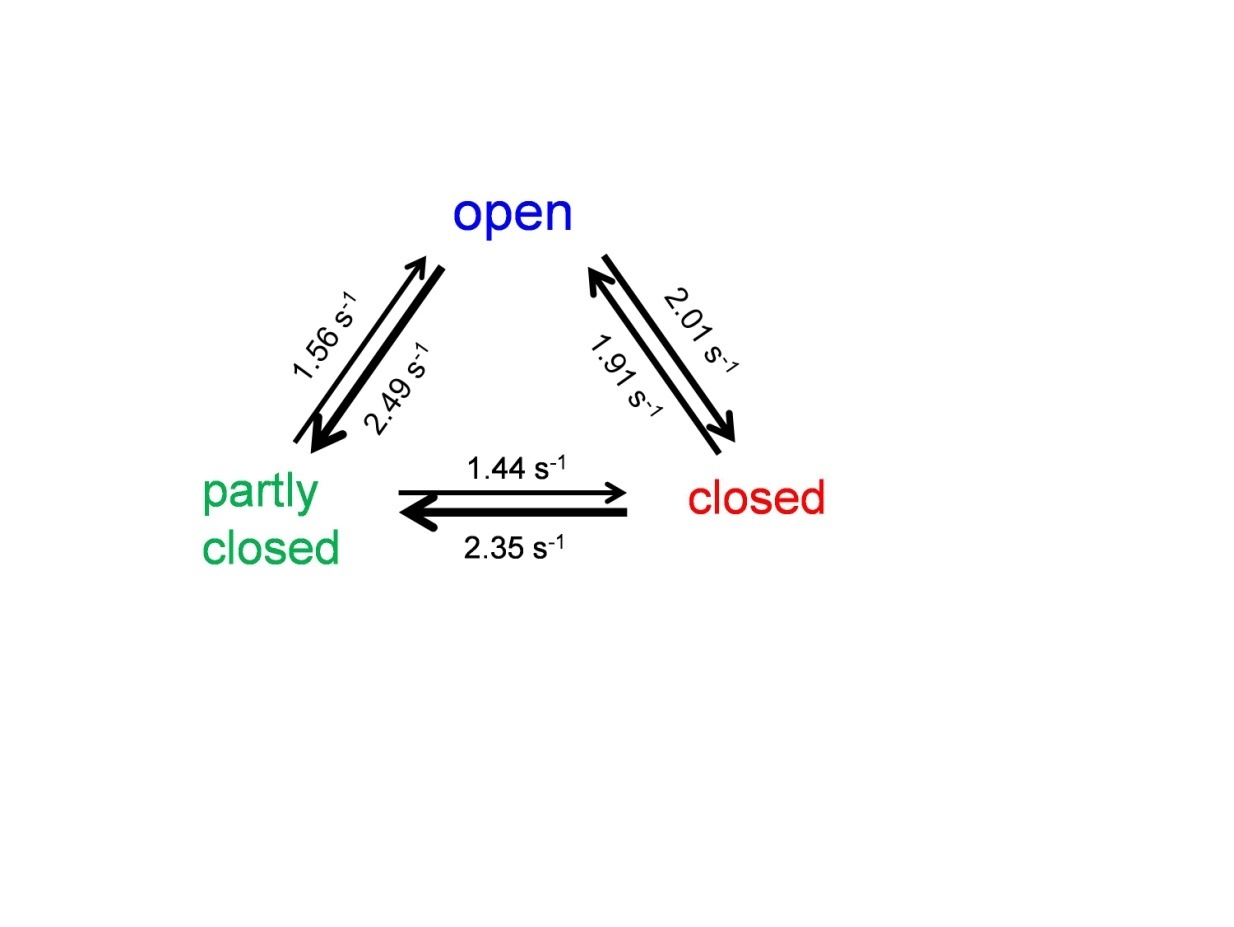

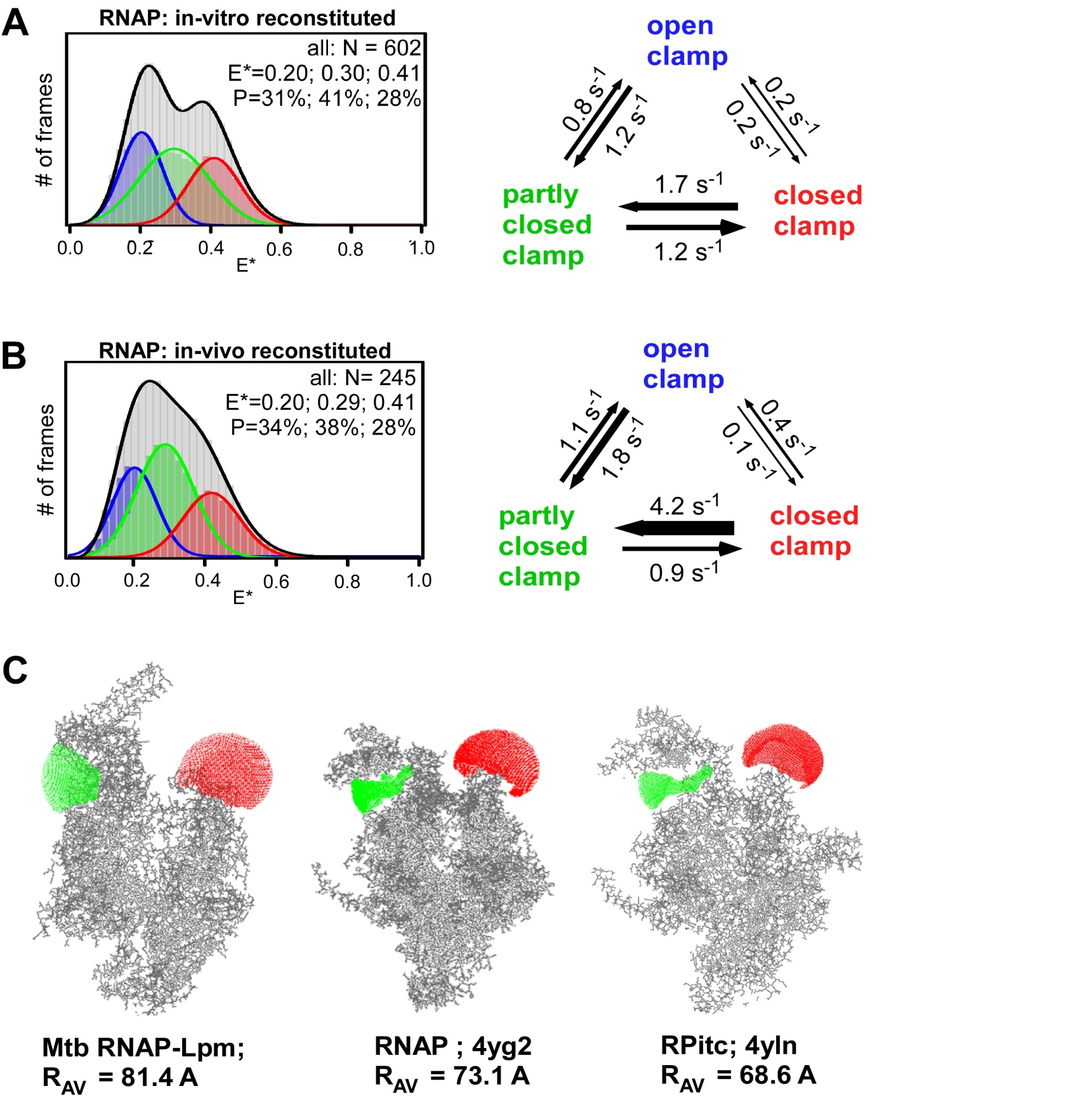
**

**B**

**A**

**B**

**
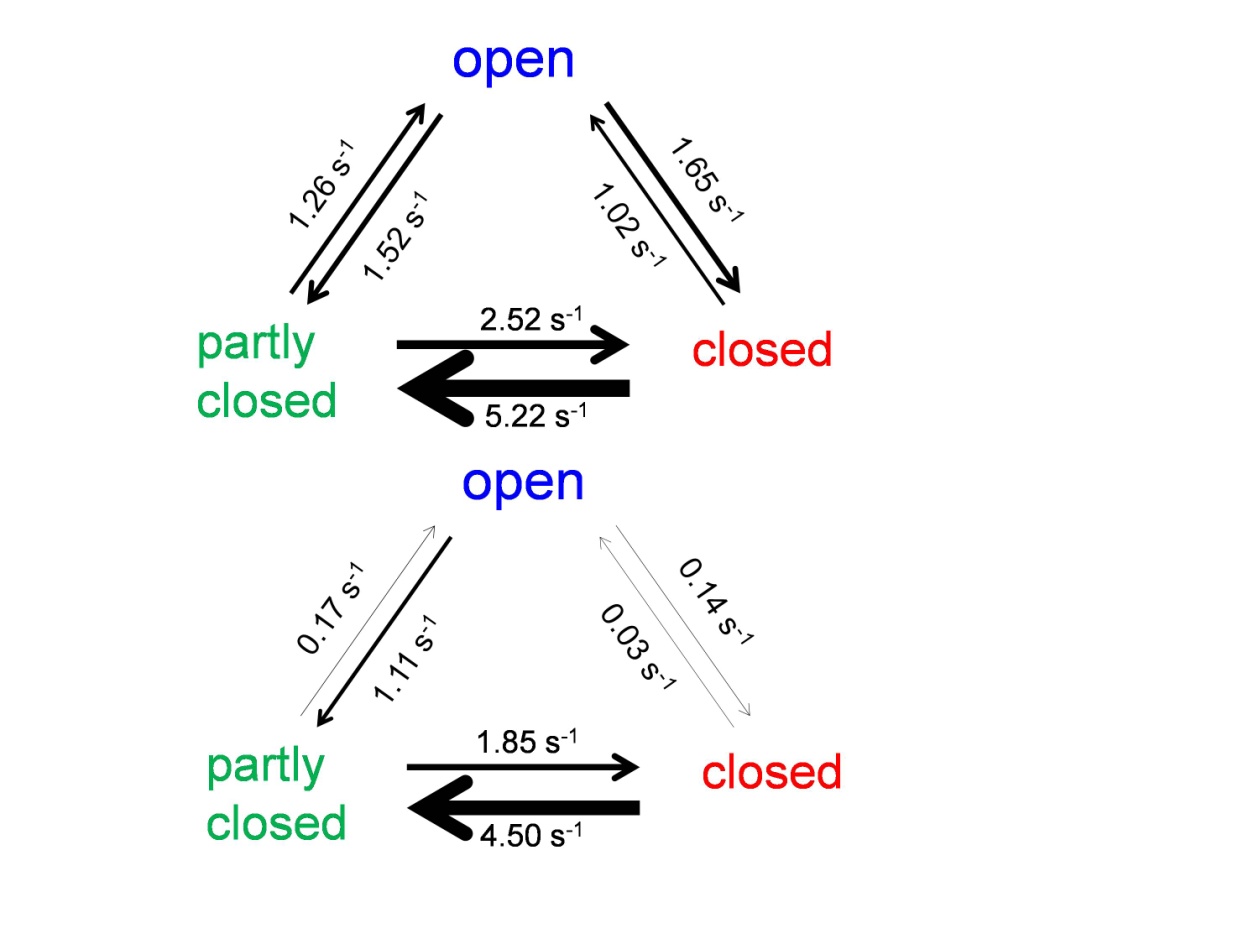
**

**C**

**
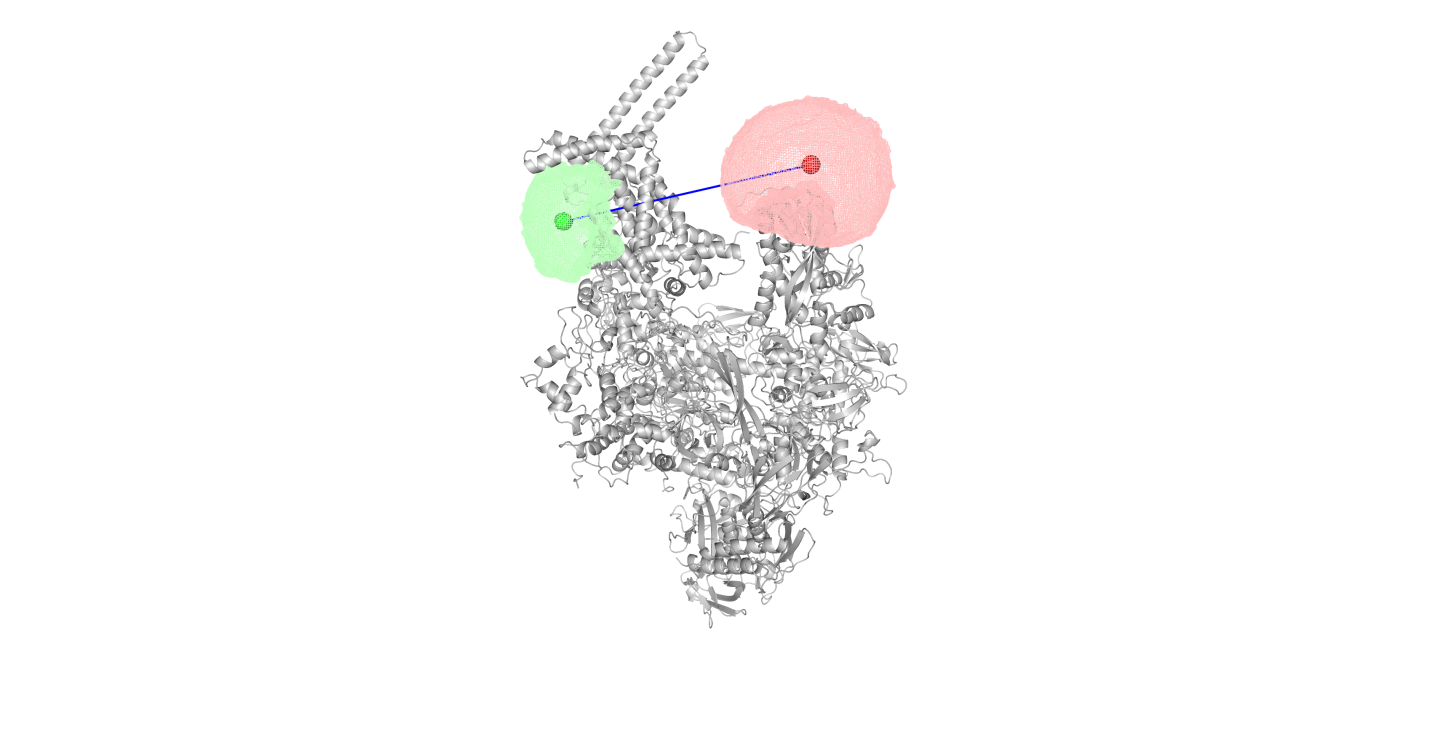
**

**
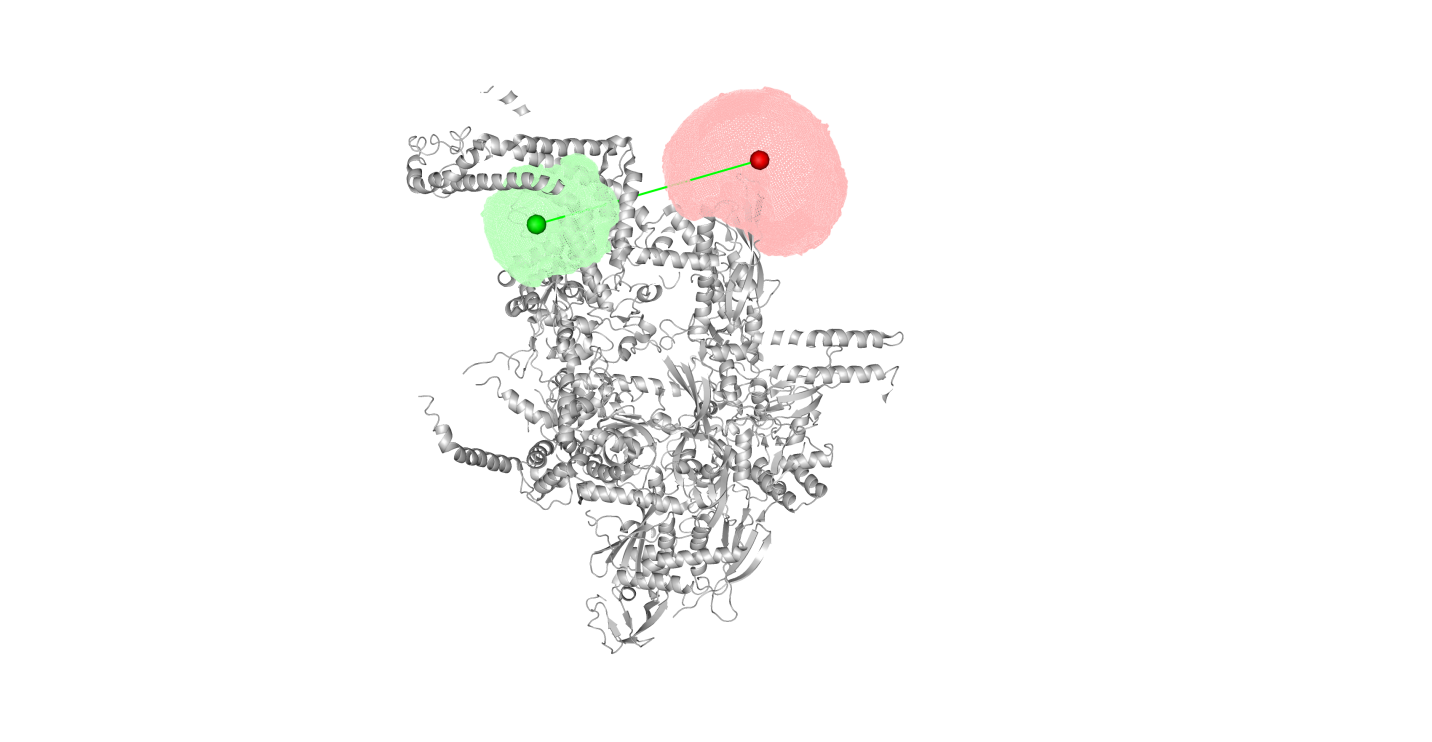

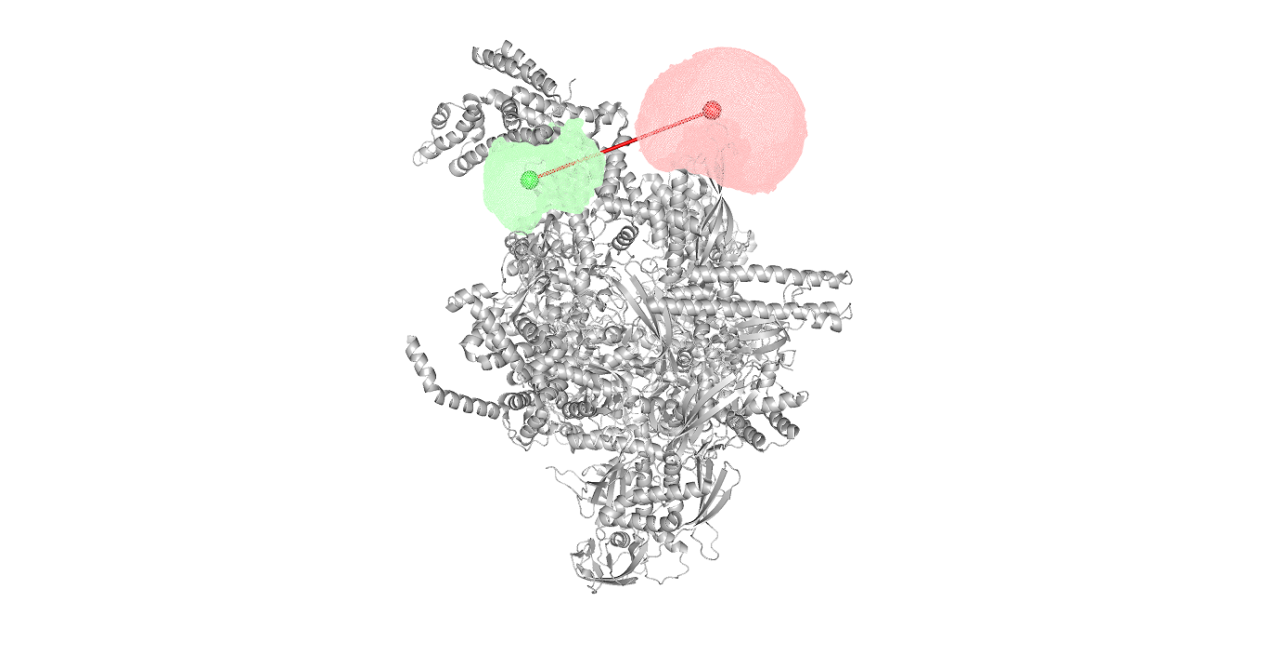
**

Mtb RNAP-Lpm RNAP holo; 4YG2 RP_ITC_; 4YLN

R_AV_ = 80 Å R_AV_ = 72.5 Å R_AV_ = 68.5 Å

**Fig. S4**


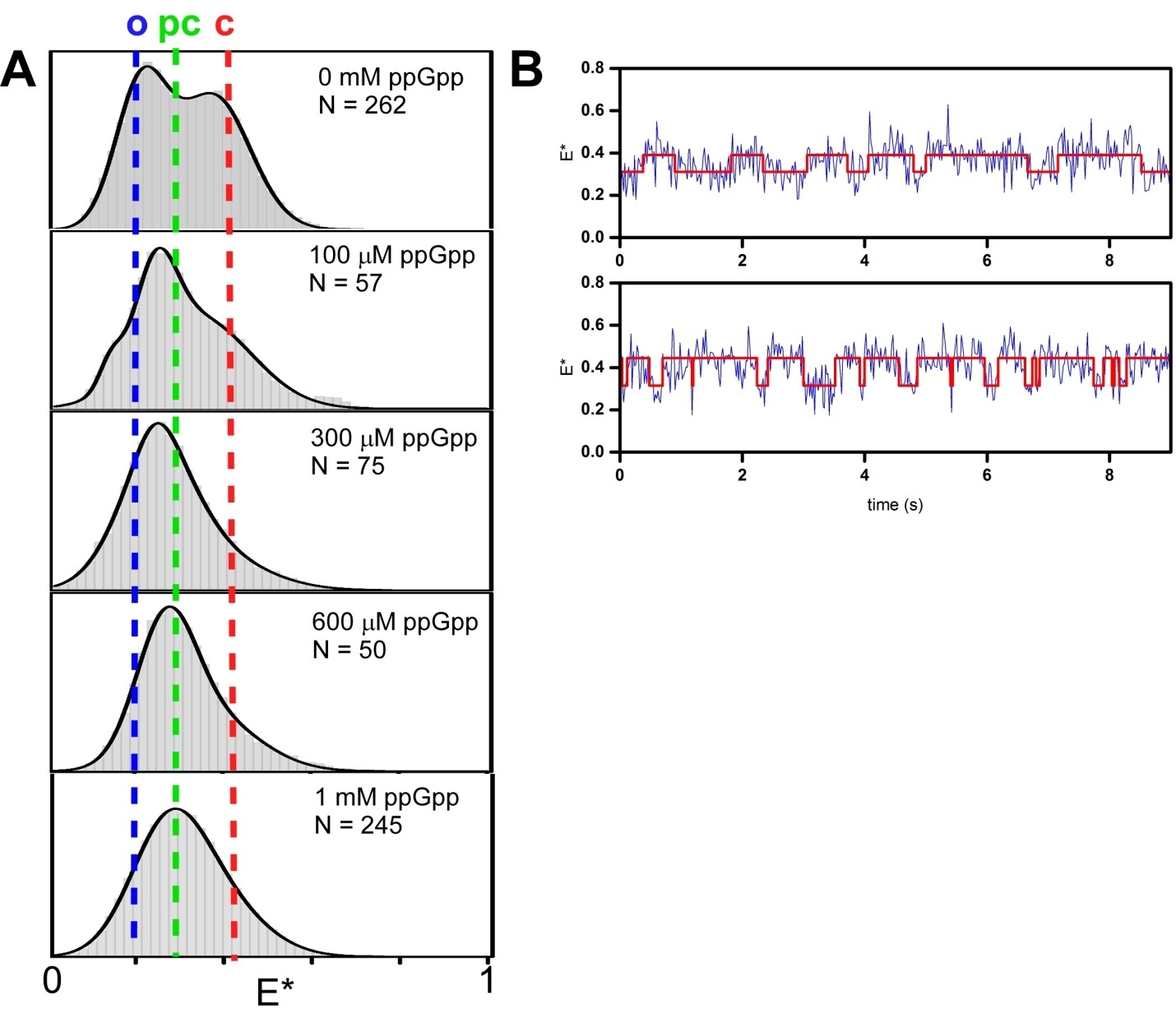


**A**

**B**

**Fig. S5**

**A**


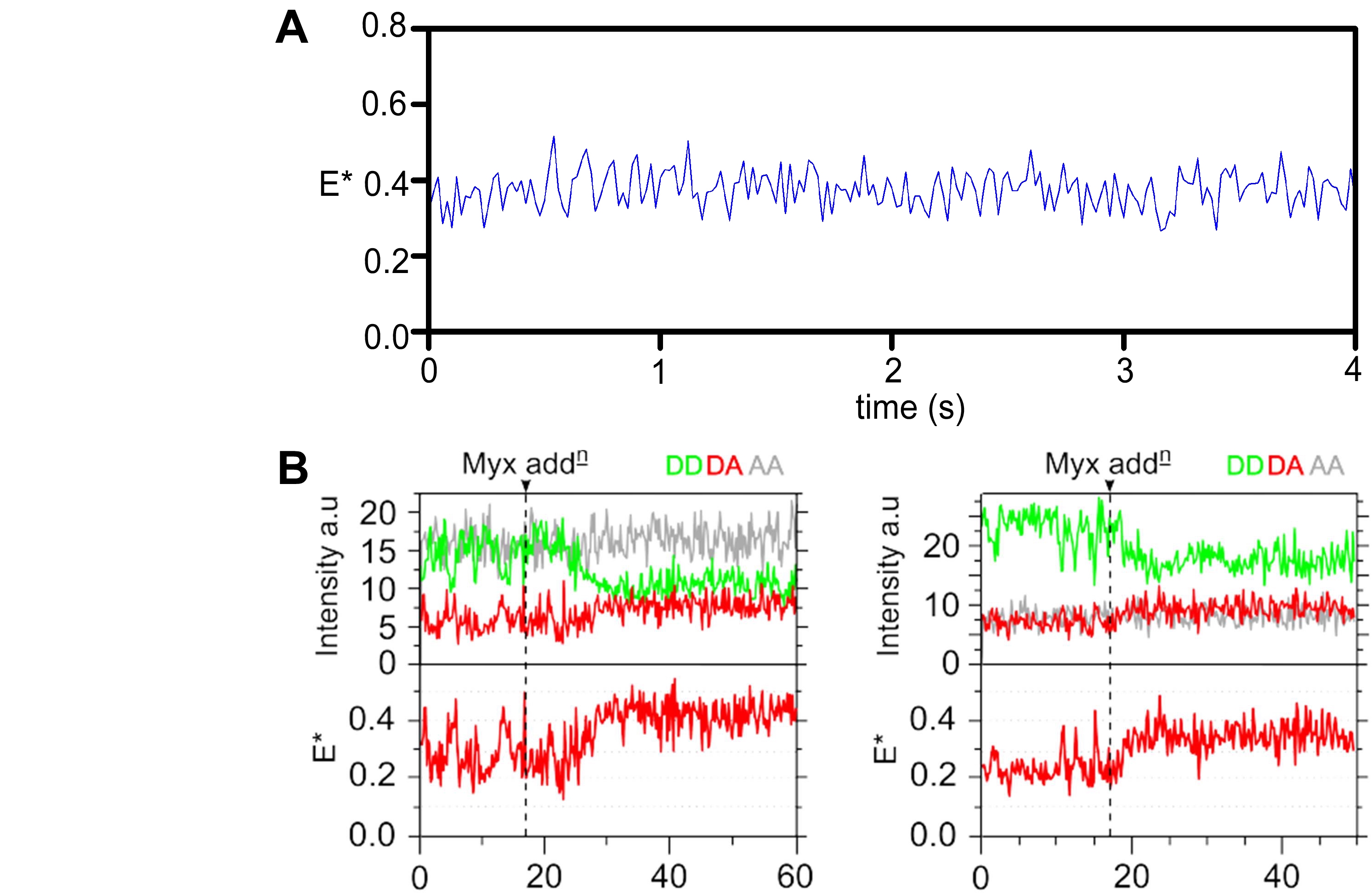

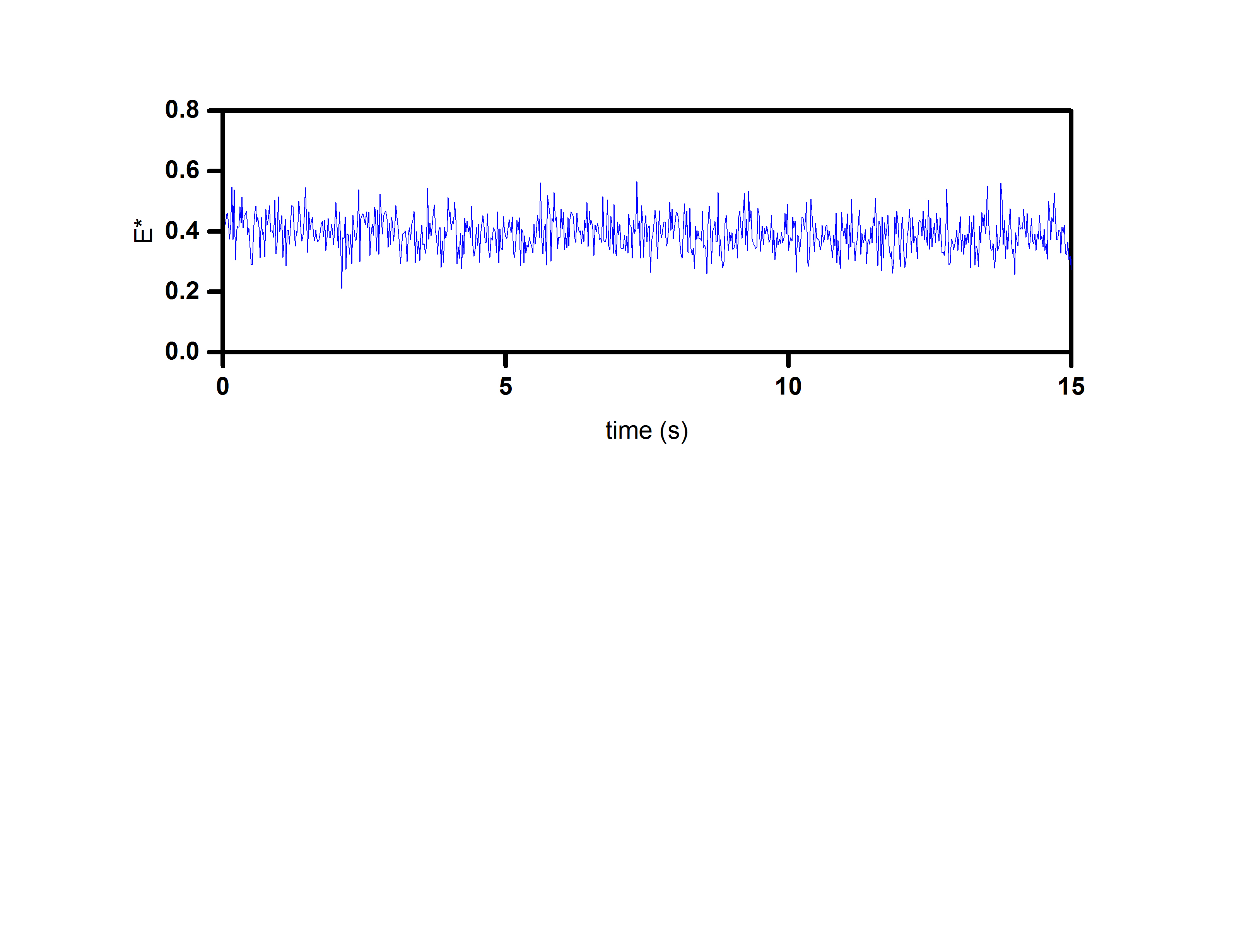

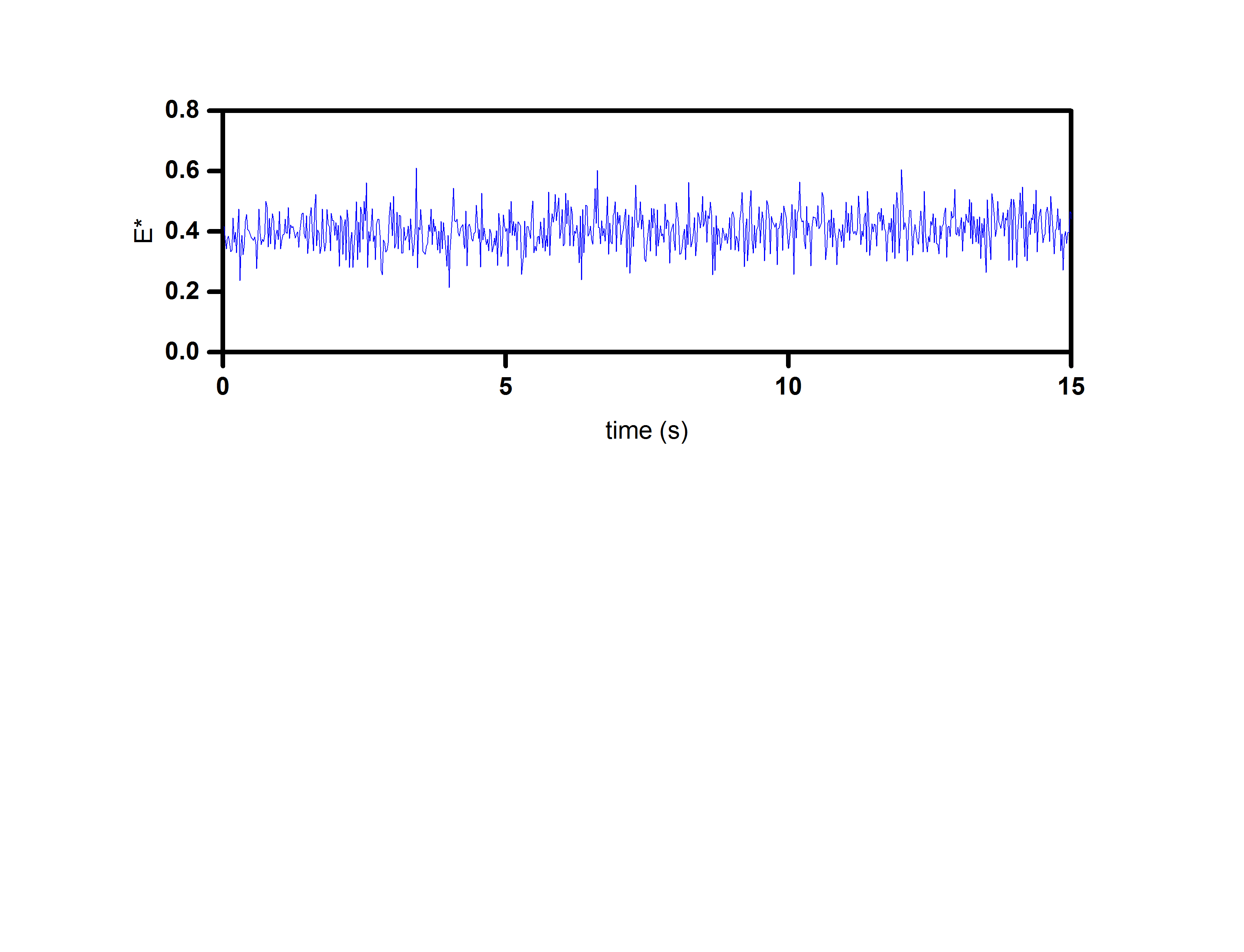


RNAP + Myx

RNAP + Myx

**A**

time (s)

time (s)

**B**

**B**

**Fig. S6**

**
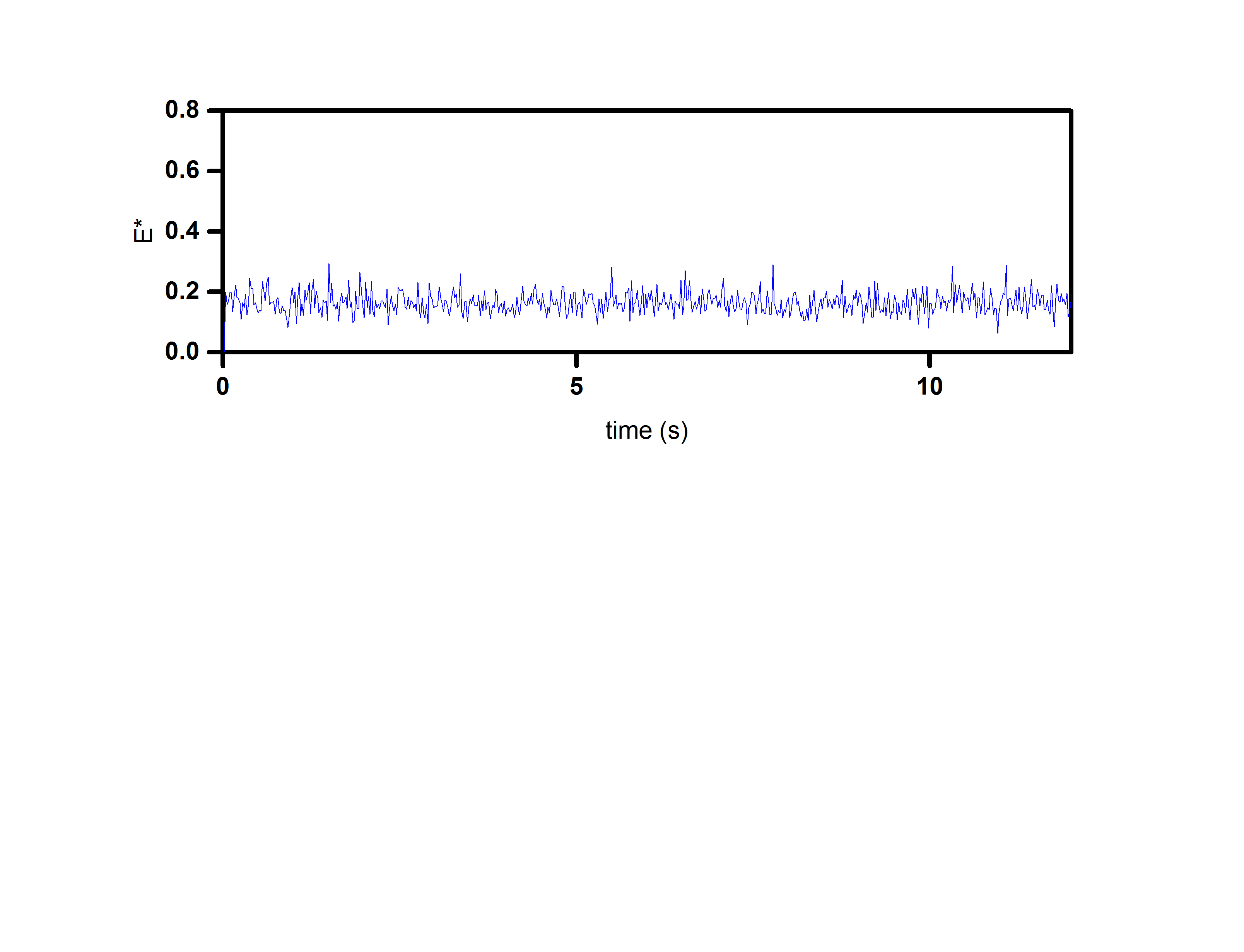

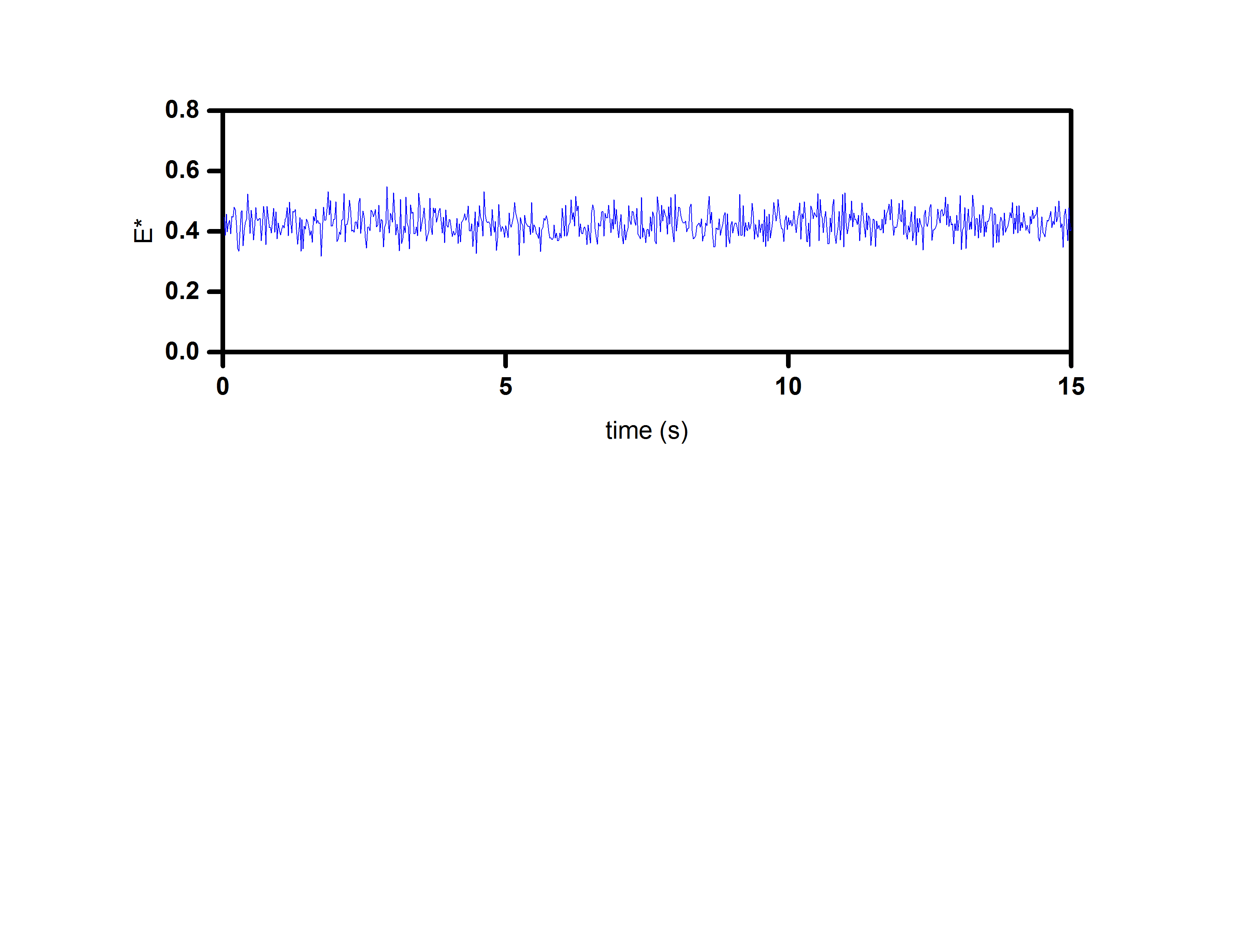
**

**A**

RPo

RPo

**
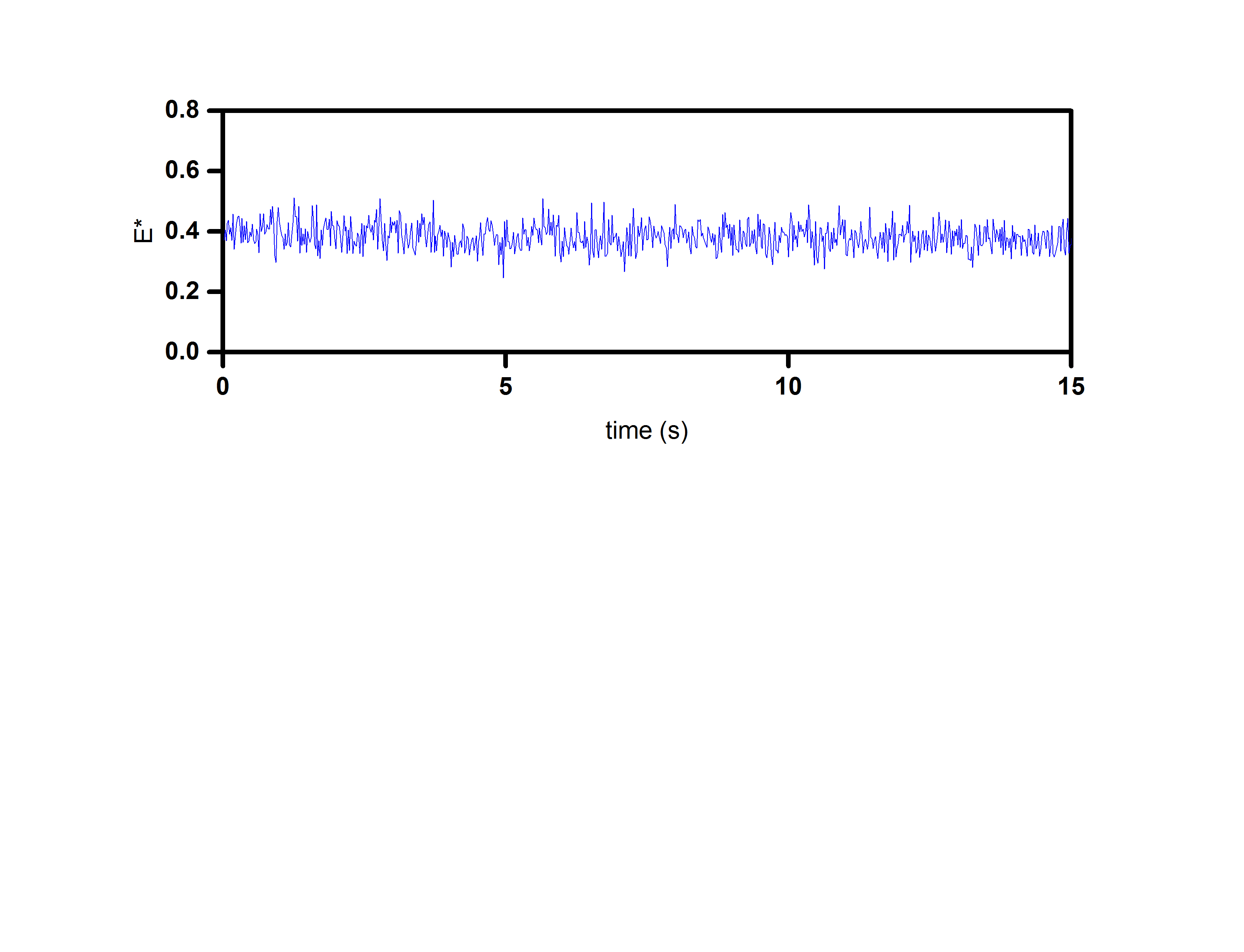

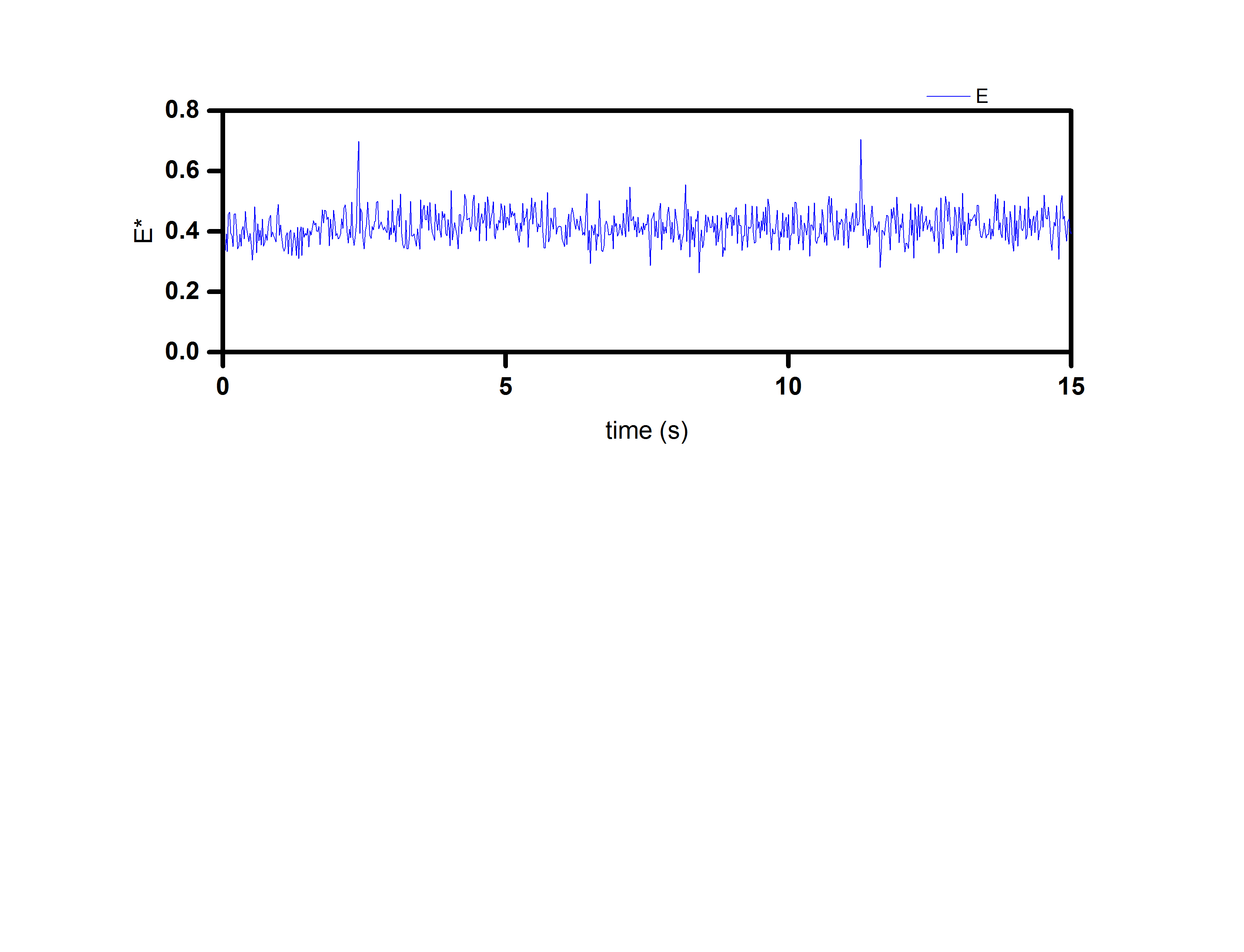
**

**B**

RPo pre-melted

RPo pre-melted

**
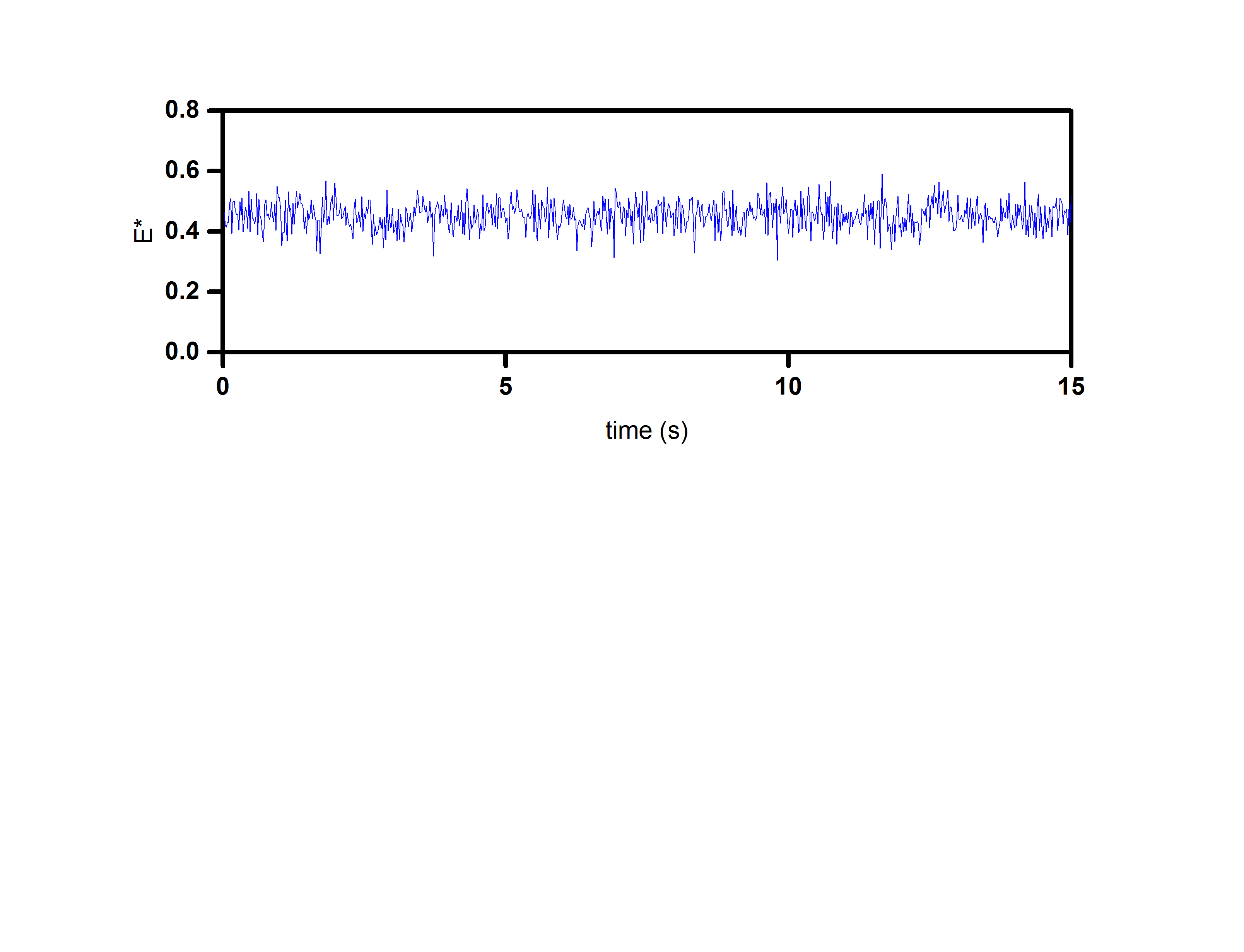

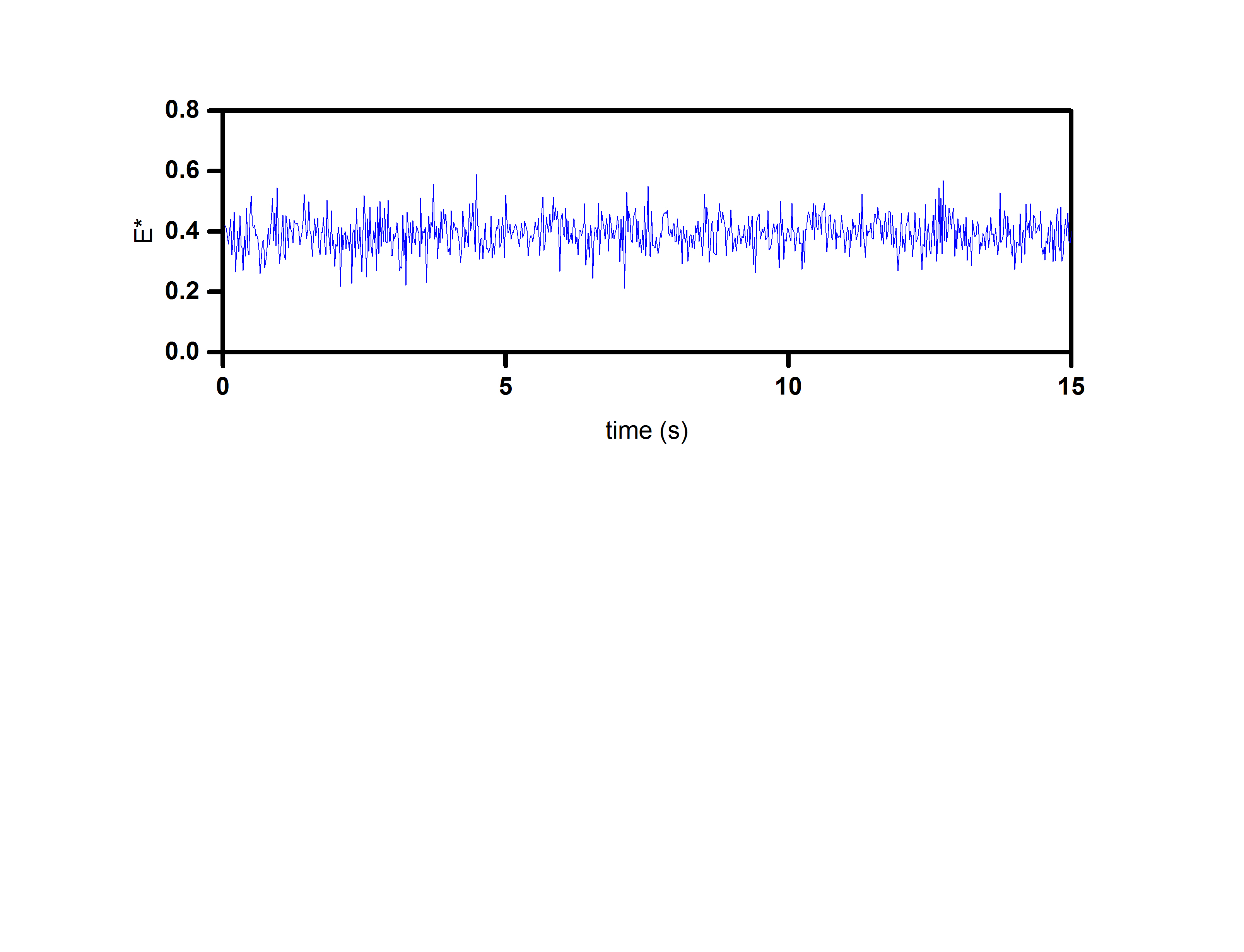
**

**C**

RDe

RDe

time (s) time (s)
